# Prediction of functional profiles of gut microbiota from 16S rRNA metagenomic data provides a more robust evaluation of gut dysbiosis occurring in Japanese type 2 diabetic patients

**DOI:** 10.3164/jcbn.17-44

**Published:** 2017-10-11

**Authors:** Ryo Inoue, Ryuji Ohue-Kitano, Takamitsu Tsukahara, Masashi Tanaka, Shinya Masuda, Takayuki Inoue, Hajime Yamakage, Toru Kusakabe, Koji Hasegawa, Akira Shimatsu, Noriko Satoh-Asahara

**Affiliations:** 1 Laboratory of Animal Science, Kyoto Prefectural University, 1-5 Shimogamo Hangi-cho, Sakyo-ku, Kyoto 606-8522, Japan; 2 Department of Endocrinology, Metabolism, and Hypertension, National Hospital Organization Kyoto Medical Center, 1-1 Fukakusa Mukaihata-cho, Fushimi-ku, Kyoto 612-8555, Japan; 3 Kyoto Institute of Nutrition & Pathology, 7-2 Furuikedani, Tachikawa, Ujitawara, Kyoto 610-0231, Japan; 4 Division of Translational Research, National Hospital Organization Kyoto Medical Center, 1-1 Fukakusa Mukaihata-cho, Fushimi-ku, Kyoto 612-8555, Japan; 5 Clinical Research Institute, National Hospital Organization Kyoto Medical Center, 1-1 Fukakusa Mukaihata-cho, Fushimi-ku, Kyoto 612-8555, Japan

**Keywords:** diabetes, dysbiosis, genus *Blautia*, glucose metabolism, prediction of functional profiles

## Abstract

We assessed whether gut microbial functional profiles predicted from 16S rRNA metagenomics differed in Japanese type 2 diabetic patients. A total of 22 Japanese subjects were recruited from our outpatient clinic in an observational study. Fecal samples were obtained from 12 control and 10 type 2 diabetic subjects. 16S rRNA metagenomic data were generated and functional profiles predicted using “Phylogenetic Investigation of Communities by Reconstruction of Unobserved States” software. We measured the parameters of glucose metabolism, gut bacterial taxonomy and functional profile, and examined the associations in a cross-sectional manner. Eleven of 288 “Kyoto Encyclopedia of Genes and Genomes” pathways were significantly enriched in diabetic patients compared with control subjects (*p*<0.05, q<0.1). The relative abundance of almost all pathways, including the *Insulin signaling pathway* and *Glycolysis/Gluconeogenesis*, showed strong, positive correlations with hemoglobin A_1c_ (HbA_1c_) and fasting plasma glucose (FPG) levels. Bacterial taxonomic analysis showed that genus *Blautia* significantly differed between groups and had negative correlations with HbA_1c_ and FPG levels. Our findings suggest a novel pathophysiological relationship between gut microbial communities and diabetes, further highlighting the significance and utility of combining prediction of functional profiles with ordinal bacterial taxonomic analysis (UMIN Clinical Trails Registry number: UMIN000026592).

## Introduction

The prevalence of type 2 diabetes has increased worldwide, including in Japan. Accumulating evidence has implicated dysbiosis of the gut microbiota in the development of type 2 diabetes.^(1)^ However, the functional profiles of the gut microbiota, including activated/inactivated biological pathways in diabetic patients, are yet to be fully elucidated.

Currently, there are two major approaches to evaluate the functions of gut microbiota by metagenomics: 1) whole-genome shotgun metagenomics and 2) computational prediction using data of 16S rRNA metagenomics,^(2)^ the former being more accurate but expensive than the latter, as it requires many more sequence reads.^(3,4)^ The latter is a recently developed approach that can predict the functions of microbial communities based solely on the computational analysis of 16S rRNA datasets, effectively eliminating the need for both prohibitively expensive metagenomic sequencing across many samples and laboratory work.^(3)^

To gain insight into the pathophysiological roles of gut microbial functions in type 2 diabetes, we examined the relationship between gut bacterial compositions, predictive functional profiles of gut microbial communities, and anthropometric/metabolic parameters in Japanese subjects with or without type 2 diabetes.

## Materials and Methods

### Subjects

A total of 22 Japanese subjects (13 men and 9 women; mean age 63.1 ± 7.6 years) were recruited from our outpatient clinic from January 2016 to February 2016. There were 12 non-diabetic control subjects and 10 type 2 diabetic patients. Details of the subjects recruiting this study were mentioned at Table 1. Patients treated with glucose-lowering agents were excluded. We measured the parameters of glucose metabolism, gut bacterial taxonomy and functional profile in all subjects without missing data, and examined the associations in a cross-sectional manner.

### Next generation sequencing

Fecal samples were collected from the subjects with a stool collection brush and storage tube (Wako Pure Chemicals, Osaka, Japan), followed by DNA extraction and 16S rRNA metagenomic sequencing using the MiSeq platform (Illumina, CA) as previously described.^(5)^ Gut bacterial composition analysis was performed as previously reported,^(5)^ except that singletons were removed in the present study.

### Data analyses

Prediction of functional profiles from 16S rRNA datasets was conducted using Phylogenetic Investigation of Communities by Reconstruction of Unobserved States (PICRUSt) software and the Kyoto Encyclopedia of Genes and Genomes (KEGG) database release 70.0, according to the tutorial on the official website (http://picrust.github.io/). The pathways involved in *Human Diseases* were removed, as these pathways apply to human cells or tissues.

Differences in the abundance of bacterial genera or KEGG pathways between groups were analyzed using STAMP software (http://kiwi.cs.dal.ca/Software/STAMP) by Welch’s *t* test with the false detection rate correction.^(5)^ Correlations of the bacterial abundance or KEGG pathways with anthropometric/metabolic parameters were examined by the Spearman’s test using R software (https://www.r-project.org/). *P*<0.05 and q-value (q) <0.1 were considered significant.

### Ethics

The study protocol was approved by the Ethics Committee for Clinical Research at the National Hospital Organization Kyoto Medical Center, and written informed consents were obtained from all participants (UMIN000026592).

## Results

A total of 22 Japanese subjects were recruited, and all of them were included in the entire cross-sectional analyses. The mean ± SD of BMI, fasting plasma glucose (FPG), and hemoglobin A_1c_ (HbA_1c_) in diabetic patients were 29.9 ± 6.6 kg/m^2^ (control: 25.6 ± 3.3 kg/m^2^), 6.9 ± 1.6 mM (control: 5.5 ± 0.6 mM), and 6.8 ± 0.8% or 50.7 ± 8.8 mmol/mol) (control: 5.6 ± 0.2% or 38.1 ± 1.9 mmol/mol), respectively (Table 1). FPG and HbA_1c_ but not BMI was significantly higher in diabetic patients than control subjects (*p*<0.01).

**Fig. 1:**
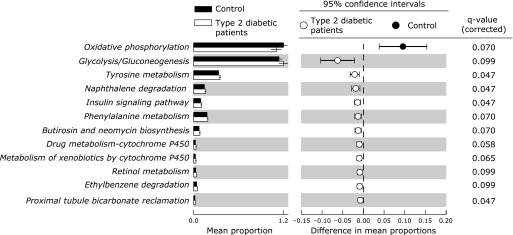
The relative abundance of functional pathways in gut microbiota between control subjects and type 2 diabetic patients. The KEGG database functional categories are shown with the displayed histograms (left panel: means ± SD) and q-value determinations (right panel: 95% confidence intervals). Black and white colors denote individual cases of control subjects and type 2 diabetic patients.

**Fig. 2:**
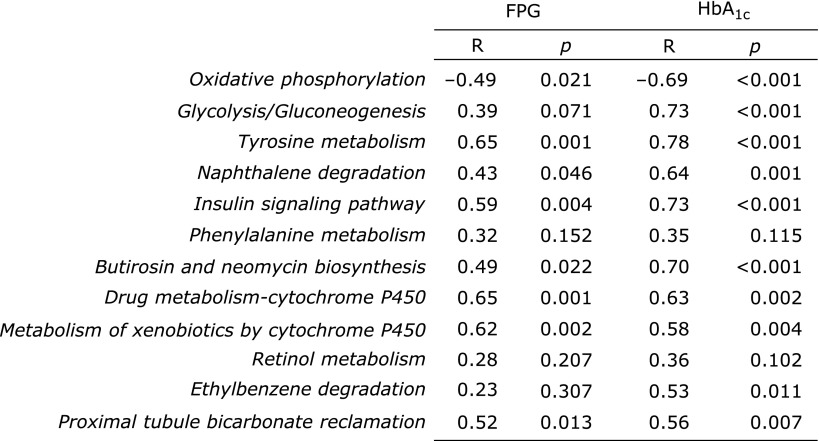
Correlations of gut microbial functional profiles with FPG and HbA_1c_ levels. Coefficient correlations (Spearman’s R) with FPG and HbA_1c_ levels, and *p* values between pairs of variables are shown respectively.

**Fig. 3:**
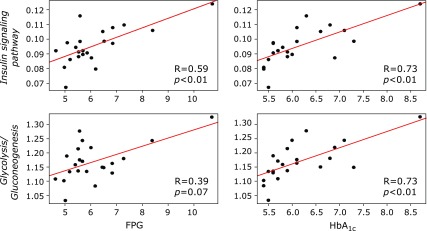
Correlations of the *Insulin signaling pathway* and *Glycolysis/Gluconeogenesis* with FPG and HbA_1c_ levels. R = Coefficient correlation (Spearman’s R). *p* = *p* value between pairs of variables.

In functional analysis, the abundance of 12 KEGG pathways was significantly different between groups (Fig. 1). The abundance of 11 pathways; *Glycolysis/Gluconeogenesis* (ko00010), *Tyrosine metabolism* (ko00350), *Naphthalene degradation* (ko00626), *Insulin signaling pathway* (ko04910), *Phenylalanine metabolism* (ko00360), *Butirosin and neomycin biosynthesis* (ko00524), *Drug metabolism-cytochrome P450* (ko00982), *Metabolism of xenobiotics by cytochrome P450* (ko00980), *Retinol metabolism* (ko00830), *Ethylbenzene degradation* (ko00642), and *Proximal tubule bicarbonate reclamation* (ko04964), were significantly higher in diabetic patients than in control subjects (*p*<0.05 and q<0.1). Conversely, the KEGG pathway *Oxidative phosphorylation* (ko00190) was the only pathway of which abundance was significantly lower in diabetic patients than in control subjects (*p*<0.05 and q<0.1). We evaluated the correlation between the abundance of these 12 KEGG pathways and the parameters of glucose metabolism (Fig. 2). The abundance of almost all pathways, especially the *Insulin signaling pathway* and *Glycolysis/Gluconeogenesis*, showed significantly strong positive correlations with HbA_1c_ (R = 0.73 and 0.73, respectively; Fig. 3). Furthermore, the *Insulin signaling pathway* showed a significantly strong positive correlation with FPG (R = 0.59), and *Glycolysis/Gluconeogenesis* tended to be positively correlated with FPG (R = 0.39) (Fig. 3).

In bacterial taxonomic analysis, only the abundance of the genus *Blautia* was significantly different between the groups (Table 2; control vs diabetic patients: 3.98 ± 2.26% vs 1.46 ± 1.38%, *p*<0.05 and q<0.1). The abundance of the genus *Blautia* showed significant negative correlations with HbA_1c_ but not with FPG levels (R = –0.63).

## Discussion

Using computational prediction from 16S rRNA metagenomic data, this study represents the first demonstration that gut microbiota-associated functional profiles in type 2 diabetic patients are remarkably different from those of control subjects. In addition, results revealed that the composition ratio of the genus *Blautia* decreased in diabetic patients compared with that in control subjects, and furthermore, negatively correlated with HbA_1c_.

It is now the world consensus that gut microbiota play an important role in obesity^(6)^ and obesity-associated disease such as type 2 diabetes.^(7)^ However, it has been difficult to clarify in detail how the gut microbiota, in particular their functional profiles, relate to pathophysiological conditions, due to the gut microbial diversity depends on individual and/or ethnic background differences.^(4)^ Recently, a few whole-genome-based shotgun metagenomics studies have suggested that functional profiles of the gut microbiota may be associated with inflammatory diseases and autoimmune diseases.^(8)^ However, using a readily available and affordable computational tool such as PICRUSt, no evidence of type 2 diabetes-associated gut microbial functional profiles has been found to date. To that end, we assessed the taxonomic and the functional profiles of gut microbiota and evaluated for the first time the correlations between these profiles and glucose metabolism in Japanese diabetic patients, using the recently developed computational prediction from 16S rRNA datasets.

Our bacterial taxonomic analysis demonstrated that the genus *Blautia* differed between control and diabetic subjects, whereas other genera were not significantly different. The genus *Blautia* is the most major bacterial group in *Clostridium coccoides*-group that was also found to decrease in Japanese patients with type 2 diabetes than control subjects.^(9)^ Accordingly, in Japanese diabetic patients, *Blautia* may be the genus of which the abundance and/or the number decrease. Further, we found that *Insulin signaling pathway* and *Glycolysis/Gluconeogenesis*, which were upregulated in diabetic patients, showed significantly strong positive correlations with HbA_1c_. Some of the genes residing in these pathways comprised the enzymes involved in degradation of carbohydrates into short-chain fatty acids (SCFAs), such as hexokinase [EC: 2.7.1.1] (K00844),^(10)^ phosphoenolpyruvate carboxykinase [EC: 4.1.1.32] (K01596)^(11)^ and acetyl-CoA synthetase [EC: 6.2.1.1] (K01895).^(11)^ Thus, these findings may imply pathophysiological alterations of SCFAs profiles in the gut of diabetic patients, as previously suggested.^(1)^ Regarding other 10 significantly different pathways, the relation with type 2 diabetes is not simply interpreted. Some of them such as *Oxidative phosphorylation*, *Tyrosine metabolism* and *Drug metabolism-cytochrome P450* suggest the alterations of bacterial metabolism. However, they are still vague to connect with type 2 diabetes. Further studies are needed to clarify the involvement of these pathways in type 2 diabetes.

Of note, we preliminary detected in nine subjects (three control and six diabetic), that gut bacterial composition and functional profiles were consistent within individuals (R = 0.90 ± 0.06 and R = 0.99 ± 0.01, respectively) throughout one month, suggesting the stability/reproducibility of this approach. Further longitudinal studies in larger cohorts with vs without dietary intervention would provide insights into a causal association between gut microbiota-functional profiles and type 2 diabetes.

In conclusion, this study provides notable evidence that not only composition but also the functional profiles of microbiota differed between Japanese control subjects and diabetic patients, which was further associated with glucose metabolism. These findings highlight the significance of identifying and characterizing the gut microbial functional pathways possibly implicated in type 2 diabetic patients, including the *Insulin signaling pathway* and *Glycolysis/Gluconeogenesis* pathways found in this study. In this regard, a recently developed, cost-effective computational prediction from 16S rRNA datasets would be helpful for a combinational evaluation of the functional and taxonomic properties of gut microbiota. Such integrated analysis of ecological and functional dynamics of microbiota would provide valuable insight into preemptive microbiome-based medicine, by identifying the intervention targets for regulating microbial function.

## Figures and Tables

**Table 1:** Patient characteristics

Variable	Control	Type 2 diabetic patients
*N* (male/female)	12 (8/4)	10 (5/5)
Age (years)	61.7 ± 6.9	64.9 ± 8.4
BMI (kg/m^2^)	25.6 ± 3.3	29.9 ± 6.6
SBP (mmHg)	133 ± 12	132 ± 15
DBP (mmHg)	81 ± 6	83 ± 10
FPG (mM)	5.5 ± 0.6	6.9 ± 1.6
HbA_1c_ (%)	5.6 ± 0.2	6.8 ± 0.8
HbA_1c_ (mmol/mol)	38.1 ± 1.9	50.7 ± 8.8
Total cholesterol (mM)	4.7 ± 0.9	4.7 ± 0.8
Triglycerides (mM)	0.9 [0.6, 1.2]	1.0 [0.8, 1.5]
HDL-cholesterol (mM)	1.8 ± 0.4	1.8 ± 1.0
LDL-cholesterol (mM)	2.5 ± 0.6	2.6 ± 0.7
Proportion (*n*, %)		
taking antidiabetic medication	0, 0.0%	5, 50.0%
taking calcium antagonist	4, 33.3%	3, 30.0%
taking ACE/ARB	3, 25.0%	2, 20.0%
taking statins	1, 8.3%	2, 20.0%

Data are expressed as the mean ± SD or median (interquartile range). BMI, body mass index; SBP, systolic blood pressure; DBP, diastolic blood pressure; FPG, fasting plasma glucose; HbA_1c_, hemoglobin A_1c_ (NGSP); HDL, high-density lipoprotein; LDL, low-density lipoprotein.

**Table 2:** Relative abundance (%) of bacterial genera in the fecal microbiota of control and type 2 diabetes subjects

Plylum	Class	Order	Family	Genus	Control	Type 2 diabetes
Actinobacteria	Actinobacteria	Bifidobacteriales	*Bifidobacteriaceae*	*Bifidobacterium*	6.55 ± 8.72	6.60 ± 6.37
Actinobacteria	Coriobacteriia	Coriobacteriales	*Coriobacteriaceae*	*Collinsella*	1.39 ± 1.37	1.20 ± 1.58
Bacteroidetes	Bacteroidia	Bacteroidales	*Bacteroidaceae*	*Bacteroides*	19.44 ± 13.24	23.52 ± 12.32
Bacteroidetes	Bacteroidia	Bacteroidales	*Porphyromonadaceae*	*Parabacteroides*	1.05 ± 1.31	0.95 ± 0.93
Bacteroidetes	Bacteroidia	Bacteroidales	*Prevotellaceae*	*Prevotella*	13.91 ± 22.06	10.84 ± 17.13
Bacteroidetes	Bacteroidia	Bacteroidales	[*Paraprevotellaceae*]	[*Prevotella*]	2.01 ± 5.30	0.07 ± 0.27
Firmicutes	Bacilli	Lactobacillales	*Streptococcaceae*	*Streptococcus*	2.53 ± 3.48	0.57 ± 0.69
Firmicutes	Clostridia	Clostridiales	unclassified	unclassified	2.93 ± 5.79	1.11 ± 1.46
Firmicutes	Clostridia	Clostridiales	*Clostridiaceae*	*Clostridium*	2.04 ± 3.54	0.93 ± 1.17
Firmicutes	Clostridia	Clostridiales	*Lachnospiraceae*	unclassified	2.37 ± 1.98	2.29 ± 2.26
Firmicutes	Clostridia	Clostridiales	*Lachnospiraceae*	Blautia*	3.98 ± 2.26	1.46 ± 1.38
Firmicutes	Clostridia	Clostridiales	*Lachnospiraceae*	*Clostridium*	1.09 ± 1.04	0.74 ± 0.73
Firmicutes	Clostridia	Clostridiales	*Lachnospiraceae*	*Coprococcus*	1.01 ± 1.34	0.47 ± 0.68
Firmicutes	Clostridia	Clostridiales	*Lachnospiraceae*	*Dorea*	2.63 ± 2.57	1.74 ± 2.44
Firmicutes	Clostridia	Clostridiales	*Lachnospiraceae*	*Lachnospira*	2.00 ± 2.55	4.04 ± 3.94
Firmicutes	Clostridia	Clostridiales	*Lachnospiraceae*	*Roseburia*	3.39 ± 3.94	4.86 ± 3.79
Firmicutes	Clostridia	Clostridiales	*Lachnospiraceae*	[*Ruminococcus*]	2.25 ± 3.15	1.03 ± 0.94
Firmicutes	Clostridia	Clostridiales	*Ruminococcaceae*	*Faecalibacterium*	5.53 ± 4.61	8.78 ± 4.97
Firmicutes	Clostridia	Clostridiales	*Ruminococcaceae*	*Gemmiger*	1.60 ± 1.68	1.54 ± 1.52
Firmicutes	Clostridia	Clostridiales	*Ruminococcaceae*	*Oscillospira*	1.50 ± 1.78	1.47 ± 0.89
Firmicutes	Clostridia	Clostridiales	*Ruminococcaceae*	*Ruminococcus*	4.61 ± 6.23	9.41 ± 5.93
Firmicutes	Clostridia	Clostridiales	*Veillonellaceae*	*Phascolarctobacterium*	1.67 ± 1.94	1.56 ± 1.57
Firmicutes	Erysipelotrichi	Erysipelotrichales	*Erysipelotrichaceae*	unclassified	1.20 ± 1.80	0.48 ± 0.79
Proteobacteria	Betaproteobacteria	Burkholderiales	*Alcaligenaceae*	*Sutterella*	1.67 ± 1.52	1.73 ± 1.29

Data are expressed as the mean ± SD. Only the taxonomy of which mean relative abundance is above 1% in the control subjects is listed. *p<0.05, q<0.1.
